# Reactive treponemal and non-treponemal tests in pregnant women and associated factors*

**DOI:** 10.1590/1980-220X-REEUSP-2022-0146en

**Published:** 2022-11-25

**Authors:** Amanda Ribeiro de Paula Reis, Geraldo Duarte, Mayra Gonçalves Menegueti, Renata Karina Reis, Ana Cláudia Rabelo e Silva, Elucir Gir

**Affiliations:** 1 Universidade de São Paulo, Ribeirão Preto, São Paulo, Brazil.

**Keywords:** Syphilis, Pregnancy, Prenatal Care, Serologic Tests, Causality, Sífilis, Embarazo, Atención Prenatal, Pruebas Serológicas, Causalidad, Sífilis, Gravidez, Cuidado Pré-Natal, Testes Sorológicos, Causalidade

## Abstract

**Objective:**

to identify the rate of reactive treponemal and non-treponemal tests in pregnant women during childbirth and to analyze the factors associated with this seroreactivity.

**Method:**

this is a cross-sectional, quantitative study with secondary sources of sociodemographic and clinical data on 2,626 pregnant women treated at a public maternity hospital in the interior of São Paulo, in 2020. For statistical analysis, Fisher’s exact test, Mann-Whitney test and the logistic regression model were used. A difference of p < 0.05 was considered statistically significant.

**Results:**

the rate of seropositivity for syphilis among pregnant women in this series was 2.74%. Among the groups with positive and non-reactive tests, marital status, occupation, place of residence and use of licit drugs indicated significant differences, but, in the final model, only unmarried marital status was associated with reactive tests (Odds Ratio: 0.169; Confidence Interval: 0.04–0.72; and p: 0.016).

**Conclusion:**

in this study, unmarried marital status was the only independent factor associated with seroreactivity for syphilis. Therefore, it is necessary to create strategies aimed at women in this condition, potentially reducing the rate of congenital syphilis.

## RESUMO

**Objetivo:**

identificar a taxa de testes treponêmicos e não treponêmicos reagentes em gestantes no período do parto e analisar os fatores associados a essa sororreatividade.

**Método:**

estudo transversal, quantitativo, com fontes secundárias de dados sociodemográficos e clínicos de 2.626 gestantes atendidas em uma maternidade pública do interior paulista, em 2020. Para a análise estatística, foram utilizados os Testes Exato de Fisher, de Mann-Whitney e o modelo de regressão logística. Considerou-se diferença com significância estatística valores de p < 0,05.

**Resultados:**

a taxa de soropositividade para sífilis entre gestantes nesta casuística foi de 2,74%. Entre os grupos com testes reagentes e não reagentes à situação maritária, ocupação, local da residência e uso de drogas lícitas indicaram diferenças significantes, mas, no modelo final, apenas a situação marital não casada apresentou associação com os testes reagentes (*Odds Ratio*: 0,169; Intervalo de Confiança: 0,04–0,72; e p: 0,016).

**Conclusão:**

neste estudo, a situação maritária não casada foi o único fator independente associado à sororreatividade para a sífilis. Por isso, faz-se necessária a criação de estratégias voltadas para mulheres nessa condição, potencialmente reduzindo a taxa de sífilis congênita.

## RESUMEN

**Objetivo:**

identificar la tasa de pruebas treponémicas y no treponémicas reactivas en gestantes durante el parto y analizar los factores asociados a esta serorreactividad.

**Método:**

estudio transversal, cuantitativo con fuentes secundarias de datos sociodemográficos y clínicos de 2.626 gestantes atendidas en una maternidad pública del interior de São Paulo, en 2020. Para el análisis estadístico, se utilizaron las pruebas exactas de Fisher, las pruebas de Mann-Whitney y el modelo de regresión logística. Se consideró estadísticamente significativa una diferencia de p < 0,05.

**Resultados:**

la tasa de seropositividad para sífilis entre las gestantes de esta serie fue de 2,74%. Entre los grupos con pruebas positivas y no reactivas, estado civil, ocupación, lugar de residencia y uso de drogas lícitas indicaron diferencias significativas, pero, en el modelo final, solo el estado civil soltero se asoció con las pruebas reactivas (odds ratio: 0,169; intervalo de confianza: 0,04-0,72; y p: 0,016).

**Conclusión:**

en este estudio, el estado civil soltero fue el único factor independiente asociado a la serorreactividad para sífilis. Por lo tanto, es necesario crear estrategias dirigidas a mujeres en esta condición, potencialmente reduciendo la tasa de sífilis congénita.

## INTRODUCTION

Syphilis is a Sexually Transmitted Infection (STIs) caused by *Treponema pallidum*, and is considered a systemic chronic infection that predominantly affects populations of regions with low purchasing power. However, it also affects people living in developing and developed countries. This fact makes syphilis a disease that concerns public health globally, mainly because it affects people throughout the life cycle, starting with congenital syphilis^(1,2)^. According to the World Health Organization (WHO), syphilis affects more than 12 million people worldwide, and its elimination remains a global challenge for health systems^(3)^.

Among the various transmission possibilities of *Treponema pallidum*, the sexual route and the transplacental route, also called vertical transmission, stand out. Based on this precept, syphilis can be acquired or congenital^(4)^.

The primary and secondary stages of syphilis represent greater risks of vertical transmission of the bacteria (around 70% to 100%), as they are disease stages with a high treponemal load^(5)^. To try to scale the problem in our country, syphilis in pregnancy was included in the list of notifiable diseases in 2005^(6)^.

Congenital syphilis has different degrees of severity, and can be asymptomatic in newborns (NB) or cause serious consequences in the short or long term, causing abortions, preterm births, fetal and neonatal deaths^(5)^. Most children with early congenital syphilis do not have symptoms at birth, so diagnosis is not always evident, and will depend on clinical suspicion for the investigation of maternal history, in addition to a detailed examination of exposed children. It is important to highlight the possibility of reinfection, if individuals are re-exposed to *Treponema pallidum*, because there is no immunization after exposure, justifying the most frequent screening in pregnancy, even in pregnant women with a previous history of syphilis^(6)^.

According to WHO global estimates in 2008, maternal syphilis and adverse pregnancy outcomes that were present occurred among women who did not have prenatal care and those who did but were not adequately tested or treated^(7)^.

Appropriate treatment is considered in pregnant women according to disease stage. In recent syphilis (primary and secondary) and in recent latent syphilis (with up to one year of evolution), benzathine penicillin of 2.4 million International Units (IU), intramuscular (IM), in a single dose should be used. To treat late latent syphilis or with manifestations of tertiary syphilis, the dose of benzathine penicillin is 2.4 million IU, IM, once a week for 3 consecutive weeks^(8)^.

For the diagnosis of syphilis, several techniques may be used, depending on disease stage. The method of direct identification of the agent is used in the primary lesion of syphilis (sample collected from the lesion), while serology is the method indicated in other disease stages, using treponemal and non-treponemal immunological tests. Currently, it is recommended to start the investigation by a treponemal test (TT), such as the rapid test or chemiluminescence, confirming with non treponemal test (NTT), such as the Venereal Disease Research Laboratory (VDRL). If the two tests are reactive, the diagnosis of syphilis is confirmed^(8)^, and it is imperative to carefully analyze clinical data, laboratory tests, history of previous infections and investigation of recent exposure. The gathering of these data allows a correct diagnosis and the definition of the appropriate treatment. This approach can only be considered completed with the correct treatment control^(6)^.

In regions where confirmatory results are inaccessible or time-consuming, treatment can be initiated even with only one reactive test, aiming to protect the fetus by preventing or treating congenital syphilis. If there are conditions to confirm the diagnosis, adequate treatment should be started immediately, regardless of gestational age, and treatment control using NTT should be monthly. The response to treatment measured by the fall in antibodies will be adequate when there is a fall in the titer by at least two dilutions up to three months after the end of treatment, three dilutions up to the 6th month and negative or stabilization of titers after the 9th month. In case of increased titration in two dilutions, absence of expected decrease in titration, persistence or recurrence of symptoms, non-adherence to treatment should be investigated or if there was no reinfection^(6,9)^.

In Brazil, there are still gaps and challenges to be faced in the elaboration and implementation of public policies in STIs. The strengthening of Primary Health Care in the comprehensive care of people with STIs and their sexual partners is essential, especially during the gestational period^(10)^.

Congenital syphilis cases can be considered a failure of the public health system to provide optimal prenatal care, as congenital syphilis can be prevented through serological screening in early prenatal care and correct treatment. Screening should be repeated around 28 ^to^ gestational week and at the time of delivery in all pregnant women whose screening tests were non-reactive. Pregnant women treated for syphilis should have their partners and NB included in this flow. Only with the implementation of health care with efficient prenatal care and effective strategies for early diagnosis and treatment of syphilis in pregnant women and partners will the reduction in the rate of congenital syphilis become a reality^(6,11,12)^.

In this context, the objective of this study is justified, in order to identify, at the time of delivery, the rate of reactive TT and NTT in pregnant women who arrive at the maternity ward. Moreover, the factors associated with the seroreactivity of these tests in pregnant women are questioned. It is noteworthy that the analysis of these factors will potentially provide subsidies for the review of prenatal programs, with emphasis on more vulnerable groups.

## METHOD

### Study Design and Location

This is a cross-sectional, retrospective study, carried out from secondary sources of information, aiming to analyze sociodemographic and clinical data of pregnant women assisted from January to December 2020 at the Reference Center for Women’s Health in Ribeirão Preto-Mater (*Centro de Referência da Saúde da Mulher de Ribeirão Preto-Mater*), a public maternity hospital in the countryside of the state of São Paulo (SP) that assists 25 other municipalities belonging to the Regional Department of Health XIII (RDH XIII)^(13)^.

### Study Population and Sample

The reference population consisted of pregnant women and their respective infants, treated at the maternity ward of a university hospital, where they underwent diagnostic tests for syphilis and delivery was performed.

In the sample, we included all parturient women and their respective infants, assisted in 2020. We excluded pregnant women who presented, during the delivery period, reactive TT and non-reactive NTT, suggesting serological memory.

To refer to the study population, two terms were used: pregnant women (since tests for syphilis diagnoses were performed before delivery or when the pregnant woman arrived at the maternity ward) and parturient women (when childbirth occurs, also mentioning the NB).

### Study Variables

In this study, the terms “reactive group” were adopted, referring to pregnant women who had reactive TT and NTT, and “non-reactive group”, referring to pregnant women with non-reactive TT.

The case definition for gestational syphilis considered the fact that pregnant women had clinical evidence and/or positive NTT with any titration and positive or not performed TT.

The dependent variable (outcome) was dichotomous (yes or no): reactive TT and NTT results. Independent or exposure variables were: maternal age; number of pregnancies/deliveries/abortions and prenatal consultations; race (white or non-white); marital status (married/in consensual union or unmarried); occupation (without formal employment or with formal employment); residence (Ribeirão Preto or other municipalities); educational level (incomplete elementary school, complete elementary school, incomplete secondary education, complete secondary education, incomplete higher education, complete higher education, graduate studies); use of licit drugs (use or not of alcohol and cigarettes); use of illicit drugs (use or not of marijuana, crack or cocaine); and pregnancy outcome (live birth or stillbirth).

The case definition of congenital syphilis considered all children born to mothers with syphilis who were not treated or were inadequately treated, plus confirmatory laboratory tests. Furthermore, when there were no conditions to serologically assess the abortion product or the dead fetus of mothers with syphilis, vertical transmission of *Treponema pallidum* ^(14)^ was considered.

The neonatal variables were the results of VDRL exams and the performance of treatment.

### Data Collection

Data collection was carried out from May to November 2021, by consulting the medical records of parturient women and their respective infants, treated at the service in the defined period.

An instrument was used for data collection, which was submitted for consideration regarding the form, content and scope of the objectives, proposed by five specialists (judges) on the subject. They were invited to sign the Informed Consent Form (ICF), after being instructed on the instrument assessment.

Data were collected from the electronic medical record system and entered using the Research Electronic Data Capture (REDCap) electronic data capture tool. This tool is a secure, web-based software platform designed to support data capture for research studies. This basis allows: 1) An intuitive interface for gathering validated data; 2) Audit trails for tracking data manipulation and export procedures; 3) Automated export procedures for seamless downloads of data to common statistical packages; and 4) Procedures for data integration and interoperability with external sources^(15,16)^.

### Statistical Analysis

The collected data were exported to a spreadsheet in Microsoft Excel^®^, with double entry, to verify eventual failures during this process and their consistency. After validating the spreadsheet, the data were imported and analyzed using the STATA SE program, version 14.

For quantitative variables, the median and maximum and minimum values were calculated. Qualitative variables were presented by frequency. To analyze the association of dependent variable with quantitative variables, the Mann-Whitney Test was used, for not following a normal distribution (assessed by the Kolmogorov-Smirnov test). To verify the association of the dependent variable with qualitative variables, Fisher’s exact test was used. Sequentially, a logistic regression model was developed. Variables with p < 0.30 in the univariate analysis were included in the final model. For all analyzes, a significance level of p < 0.05 was adopted.

In this study, the seroreactivity rate for syphilis was calculated, considering the total number of pregnant women treated during the period. It was not possible to calculate the prevalence in the population assessed, as the denominator was not known, which refers to the universe of pregnant women in the communities from which they come.

### Ethical Aspects

The present study was carried out in accordance with the ethical precepts that involve research with human beings, in accordance with Resolution 466/2012. The study was approved in 2020 by the institution’s Research Committee (Opinion 004/2020), for data collection in medical records referring to the information of parturient women in 2019; later, the request for data collection of parturient women treated in 2020 was changed. The project was approved by two other Research Ethics Committees (REC), that of the *Universidade de São Paulo* at *Escola de Enfermagem de Ribeirão Preto* (Opinion 4,277,413, in 2020, and Opinion 4,649,267, in 2021) and that of the co-participating institution (general hospital to which the maternity unit is linked, with Opinion 4,303,296, in 2020, and Opinion 4,657,861, in 2021).

The waiver of the ICF was requested and authorized in relation to pregnant women’s data, based on the impossibility and impossibility of individual/personal access or via telephone to participants, in order to invite them to participate in the research, since the study refers to a retrospective assessment.

## RESULTS

We included 2,675 parturient women treated at the study maternity hospital and their respective infants, from January to December 2020. We excluded 49 parturient women from the study, as they had positive TT and non-reactive NTT during delivery, suggesting serological memory. Thus, data from 2,626 parturient women and their NBs were analyzed. Of these, 2,554 (97.26%) were pregnant women who, during the delivery period, had non-reactive TT and 72 (2.74%) had both TT and NTT seroreactive, resulting in a seroreactivity rate for syphilis of 2.74%. Table 1 shows the data with some cracteristics of these pregnant women.

**Table 1.** Comparison of the group of pregnant women who are non-reactive to the non-treponemal test with the group who are reactive to the treponemal tests and non-treponemal tests that arrive at the maternity hospital in relation to age and gestational history – Ribeirão Preto (SP), Brazil, 2020.

Pregnant woman group	Non-reactive TT* (n = 2,554)			Reactive TT and NTT (n = 72)			p-value*
Quantitative variables	Median	Minimum value	Maximum value	Median	Minimum value	Maximum value	
Maternal age	25	13	47	23	14	39	0.06
Number of pregnancies	2	1	7	3	1	7	0.32
Number of births	1	0	6	1	0	5	0.28
Number of miscarriages	1	1	5	1	1	3	0.29
Number of prenatal consultations	9	2	10	9	2	10	0.41

*Mann-Whitney test; TT – treponemal test; NTT – non-treponemal test. Source: the author.

No differences were found between the groups for the quantitative variables: maternal age, number of pregnancies/births/miscarriages and prenatal consultations.

According to the data presented in Table 2, in relation to the marital status, in which women reported whether they were married or not, unmarried women had a higher frequency of reactive tests compared to married women (p < 0.000).

**Table 2.** Comparison of the non-reactive group and the reactive group arriving at the maternity hospital according to sociodemographic data – Ribeirão Preto (SP), Brazil, 2020.

Pregnant woman group	Non-reactive TT*		Reactive TT and NTT		Total		p-value*
Qualitative variables	nº	(%)	nº	(%)	nº	(%)	
**Race**							
White	1504	97.66	36	2.34	1540	100	0.146
Non-white	1050	96.69	36	3.31	1086	100	
**Marital status**							
Unmarried	1959	96.60	69	3.40	2028	100	0.000
Married	594	99.50	3	0.50	597	100	
Not recorded	1	100	0	0.00	1	100	
**Occupation**							
No formal job	1433	96.50	52	3.50	1485	100	0.019
With formal job	987	98.11	19	1.89	1006	100	
Not recorded	134	99.26	1	0.74	135	100	
**Residence**							
Ribeirão Preto	1314	96.48	48	3.52	1362	100	0.012
Other municipalities	1240	98.10	24	1.90	1264	100	
**Educational level**							
Incomplete elementary school	531	94.99	28	5.01	559	100	0.002
Complete elementary school	246	96.85	8	3.15	254	100	
Incomplete high school	546	96.81	18	3.19	564	100	
Complete high school	1096	98.38	18	1.62	1114	100	
Complete higher education	118	100	0	0.00	118	100	
Graduate education	5	100	0	0.00	5	100	
Not recorded	12	100	0	0.00	12	100	
**Licit drug use**							
No	1468	97.80	33	2.20	1501	100	0.035
Yes	201	95.26	10	4.74	211	100	
Not recorded	885	96.80	29	3.20	914	100	
**Illicit drug use**							
Yes	36	94.74	2	5.26	38	100	–
Not recorded	2518	97.30	70	2.70	2588	100	
**Pregnancy outcome**							
Live births	2535	97.31	70	2.69	2605	100	0.311
Stillbirth	19	90.48	2	9.52	21	100	

*Fisher’s exact test; TT – treponemal test; NTT – non-treponemal test. Source: the author.

To assess the predictive variables of pregnant women who arrive at the maternity hospital with reactive TT and NTT, a logistic regression model was used (Table 3). The final regression model showed that only the marital status (OR: 0.169; CI (0.04–0.72) and p: 0.016) was associated with the occurrence of pregnant women arriving at the maternity hospital with reactive TT and NTT. Thus, being married is a protective factor, and this group is less likely to have reactive TT and NTT compared to unmarried women.

**Table 3.** Predictive variables of the sample of pregnant women who arrive at the maternity hospital with reactive treponemal and non-treponemal tests – Ribeirão Preto (SP), Brazil, 2020.

Predictor variables/parameters	Odds Ratio (OR)	Standard error	Confidence Interval (95% CI)	p-value*
Maternal age	0.988	0.037	(0.93 – 1.05)	0.716
Number of births	1.051	0.158	(0.78 – 1.41)	0.736
Number of miscarriages	1.421	0.373	(0.85 – 2.38)	0.181
Categorized race	1.472	0.467	(0.79 – 2.74)	0.222
Categorized marital status	0.169	0.125	(0.04 – 0.72)	0.016
Categorized occupation	0.890	0.305	(0.45 – 1.75)	0.734
Categorized residence	0.597	0.194	(0.32 – 1.13)	0.113
Educational level	0.859	0.109	(0.01 – 2.96)	0.232
Categorized licit drug use	1.583	0.613	(0.74 – 3.38)	0.235

*Logistic regression. Source: the author.

There were 42 NB with VDRL performed in the blood that presented reactive results and 25 with exam performed in CSF and non-reactive results. Of the 42 NB with reactive results, 17 underwent treatment with penicillin. Moreover, another 4 NB were treated, even though the NB had a negative test.

## DISCUSSION

Of the 2,626 pregnant women who participated in this study, 72 were diagnosed with syphilis, resulting in a seroreactivity rate of 2.74% for this disease. In a national-based study assessing parturient women assisted in maternity units of the Unified Health System (SUS – *Sistema Único de Saúde*) and affiliated ones, Cunha & Merchand-Hamann^(17)^ cite different rates of syphilis prevalence considering the various regions of Brazil. The overall prevalence of syphilis in the country was estimated at 0.89%, varying between the North (1.05%), Northeast (1.14%), Southeast (0.73%), South (0.48%) and Midwest (1.20%). Therefore, they are much lower than those reported in our sample.

On the other hand, an investigation carried out in a capital in northeastern Brazil from June to September 2010, enrolled 222 parturient women, of which 17 women (7.7%) had a positive VDRL. This study identified a high prevalence of syphilis in parturient women in this region, highlighting that this situation is associated with sociodemographic, behavioral and institutional variables^(18)^.

Another study, carried out in Spain from 2002 to 2010, showed a much lower rate than that of the present study, with 85,806 pregnant women, 94 of whom were diagnosed with syphilis, with a prevalence of syphilis in pregnancy of 0.11%^(11)^. This low rate of gestational syphilis can be explained by the fact that it is a country with more advanced health policies.

Analyzing the variables individually, all quantitative variables, such as the age of pregnant women and those related to the gestational history (the number of pregnancies, deliveries, micarriages and prenatal consultations) did not show differences between the groups.

Regarding age, also in another study, it was identified that the age group was not associated with syphilis in pregnant women, sociodemographic, behavioral and health care factors were associated with the occurrence of syphilis in pregnant women, and should be considered in the development of strategies for the prevention and elimination of syphilis, focusing on situations of greater vulnerability^(19)^.

A study conducted using public data registered in the Brazilian Live Birth Information System (SINASC – *Sistema Brasileiro de Informações sobre Nascidos Vivos*), 2016, sought to assess the adequacy of prenatal care offered in Brazilian capitals and the diagnosis of gestational syphilis. Prenatal care inadequacy was associated with women under 20 years of age and with less than 4 years of education. According to the same study, only 1.3% of women did not perform prenatal care and, when performed properly, was significantly lower for black adolescents, with low education, women belonging to social classes D and E, women without work and women without a fixed sexual partner^(20)^.

In this study, no statistical differences were observed in relation to the number of pregnancies, deliveries and abortions, unlike another study, in which it was found that there is a greater risk of occurrence of syphilis among multiparous women (OR: 2.2; Confidence Interval: 1.3–3.9)^(19)^.

Regarding the number of prenatal consultations, in the present investigation, no statistical differences were detected, differing from a research that identified that syphilis in parturient women was associated with non-performance of prenatal care, fewer consultations and late initiation of prenatal care. Of the parturient women diagnosed with syphilis during prenatal care, 53.1% remained infected at birth^(17)^. In the logistic regression analysis performed in another study, one of the determining factors for gestational syphilis was the occurrence of only one to three prenatal consultations (OR: 3.5) and previous history of STI (OR: 9.7)^(19)^.

Another study, carried out in northeastern Brazil, from 2007 to 2017, identified in multivariate analyzes that prenatal coverage was significantly associated with the incidence rate of gestational syphilis, but not with the rate of congenital syphilis. The authors also point out that, even with the increase in prenatal coverage and improvement in the identification of syphilis cases in pregnant women, there was no reduction in the rate of congenital syphilis, indicating the possibility that some syphilitic pregnant women did not receive adequate treatment to prevent vertical transmission. This situation may be associated with late initiation of prenatal care and delay in test results, which may result in late diagnosis and inadequate treatment of pregnant women^(21)^.

An integrative review, assessing articles published in the period from 2010 to 2018, pointed out non-white pregnant women as factors related to the vertical transmission of syphilis, aged between 20 and 29 years, with low socioeconomic status, single and who were not treated or treated inadequately. The study also highlights that there was an increase in cases of congenital syphilis due to inadequate care, such as not performing serological tests in pregnant women and sexual partners, resulting in non-treatment of both, in addition to limited information from health professionals to pregnant women and their partners^(22)^.

Regarding the qualitative variables, also analyzed individually, unmarried marital status, occupation without formal employment, place of residence in Ribeirão Preto and licit drug use showed significant differences associated with the reactive group. On the other hand, race and pregnancy outcome did not present significant differences between groups.

In relation to race and education, the result of this study differs from a study in which parturient women of yellow, black and brown race/color and those with less education were more likely to be diagnosed with syphilis than those of race/color white and those with higher education^(17)^.

In the present sample, considering the multivariate analysis by the logistic regression model, the factor associated with the seroreactivity of TT and NTT in pregnant women at the time of delivery was the unmarried marital status. The secondary data obtained does not include the number of partners of pregnant women. Literature data indicate that women with three or more different sexual partners in the last year were three times more at risk for syphilis, that is, the number of sexual partners was associated with the outcome for increased syphilis rates^(19)^.

In another study, according to unadjusted analysis, there was a significant positive association between syphilis and being from Fortaleza, having studied for less than nine years, having had more than one sexual partner in life, not living with a partner, and illicit drug use by parturient women and by their partners. However, data analysis by adjusted multiple logistic regression did not show statistical significance for any of the variables studied^(18)^.

Regarding occupation, a survey that analyzed the epidemiological profile of cases of congenital syphilis in the city of Sobral (Ceará) showed that the occurrence of these cases was notably related to failures in treatment of infected pregnant women, indicating the need for better prenatal strategies to prevent the disease. Preventive actions in Primary Care Health services should seek early detection of pregnant women’s diagnosis and effective treatment as well as that of their partners. This should be addressed from the beginning of their sexual partner’s pregnancy, raising awareness of the risk that syphilis can bring to children. The research also identified, unlike the study in question, that the disease affects socially less favored mothers, with low education and, generally, having the occupation of being a housewife^(23)^.

In an updated review of the epidemiology, diagnosis, and treatment of gonorrhea, chlamydia, syphilis, Mycoplasma genitalium, trichomoniasis, and genital herpes, from 2015 to 2019, rates of gonorrhea, chlamydia, and syphilis are reported to have increased in the USA, while from 1999 to 2016, rates of herpes simplex virus types 1 and 2 (HSV-1 and HSV-2) have decreased. About 1 in 5 adults in the USA acquired an STI in 2018. The group with the highest rates of STIs is characterized by people under age 25, sexual and gender minorities such as trans men and women who have sex with men, and racial and ethnic minorities such as blacks and Latinos. STIs are associated with the acquisition and transmission of HIV and are the leading cause of tubal factor infertility in women. Serology continues to be recommended to diagnose syphilis, usually with the use of sequential tests to detect treponemal and non-treponemal antibodies, and benzathine penicillin was the medication used for the correct treatment of syphilis. Effective interventions for STI prevention include screening, contact tracing of sexual partners, and promotion of effective barrier contraception^(24)^.

Research developed in Fortaleza analyzed 175 reported cases of syphilis in pregnant women, compared to notifications of congenital syphilis from 2008 to 2010, and the consequences for fetuses and NBs. The results showed that more than 85% of cases had inadequate treatment, 62.9% of sexual partners were not treated or the information was ignored, and, among the pregnancies, five were stillbirths, one was a miscarriage and three were neonatal deaths. Even though most pregnant women were diagnosed with syphilis during prenatal care, congenital syphilis still occurred, which shows failure in prenatal care and late diagnosis, as most notifications occurred between the second and third trimesters of pregnancy. Routine prenatal exams oriented by the Ministry of Health to investigate neurosyphilis were not performed in NB and a good part of results of stillbirth, infant death and abortion could have been avoided with adequate care of pregnant women and early diagnosis^(25)^.

Since 2007, who has proposed global strategies to eliminate congenital syphilis^(26)^. A study shows that most countries in Latin America and the Caribbean use protocols and strategic plans, making progress towards the elimination of congenital syphilis, but they still need to improve the collection of information on prenatal coverage, mainly in relation to diagnosis and treatment and use them in decision-making, reinforcing good practices^(27)^.

Even though it is not an easy goal to achieve, some countries have shown the opposite, as, in 2015, Cuba was the first country in the world to meet the goals and be validated by the WHO for having eliminated vertical transmission of HIV and syphilis. Even with limited financial resources, success in achieving this goal is achieved through solid Primary Health Care infrastructure, effective data information systems, and well-trained medical and nursing professionals providing affordable basic health services free of charge to all. Thailand, Belarus and the Republic of Moldova also achieved validation of elimination of mother-to-child transmission of syphilis in June 2016^(26,28)^. In Brazil, in 2021, Boa Vista da Aparecida (Paraná) also achieved validation of the elimination of vertical transmission of HIV and syphilis^(29)^. Therefore, it is possible to eliminate congenital syphilis, with the application of strategies aimed at the results of studies that, for the most part, are already existing problems and that prevent this eradication.

The study presented as a limitation the non-generalization, since the data encompass a single maternity hospital. It is important to highlight, however, that the results obtained bring effective contributions, since the place is a reference maternity hospital for the SUS and that it serves women from 25 other municipalities.

## CONCLUSION

Syphilis continues to affect pregnant women, despite being subject to adequate prevention, diagnosis and treatment. In this study, it is evident that there are still failures that occurred during prenatal care, allowing undiagnosed pregnant women, consequently without treatment, to arrive at the time of delivery with a seroreactivity rate of 2.74% for this disease. Unmarried marital status was the independent factor associated with seroreactivity for syphilis. Therefore, to reduce the possibility of pregnant women arriving at the maternity hospital with reactive TT and NTT and, consequently, preventing the vertical transmission of syphilis, it is necessary to create awareness strategies on testing and treatment of the disease for pregnant women and their partners, especially for pregnant women in this marital status. Additionally, data indicated the need to improve prenatal care quality both for screening and for adequate diagnosis and treatment.
